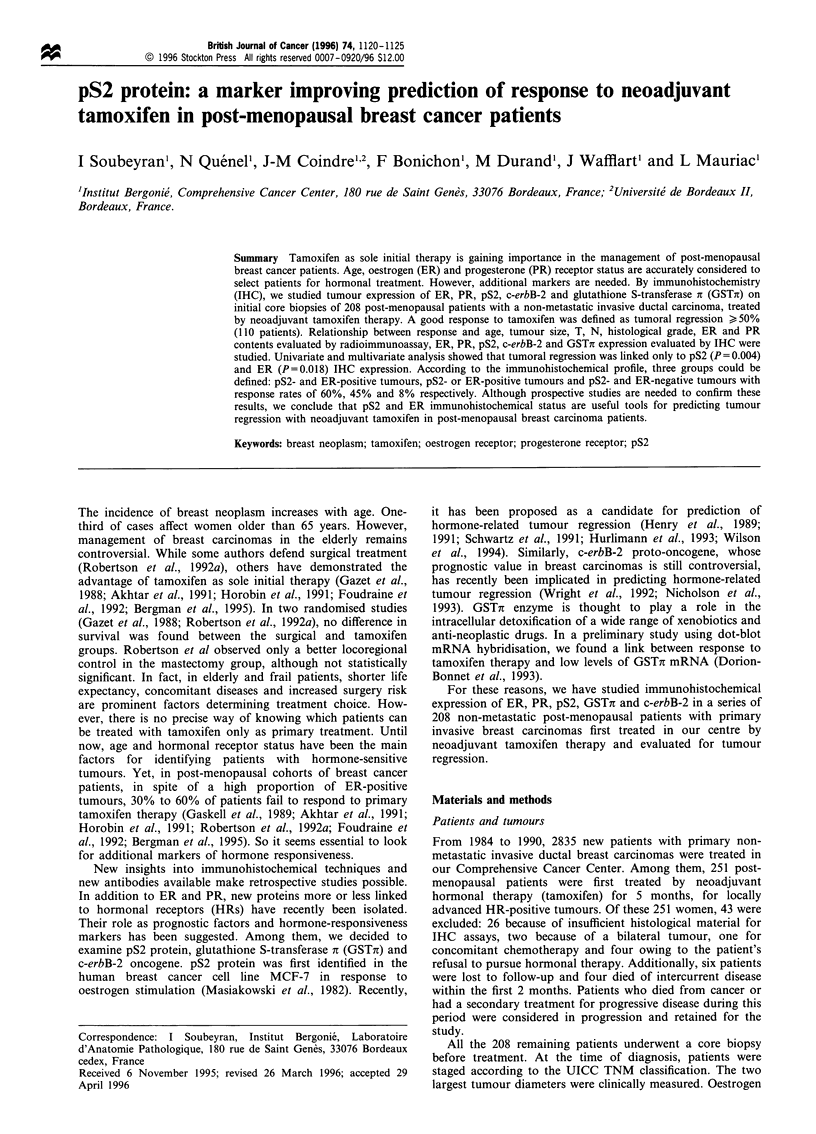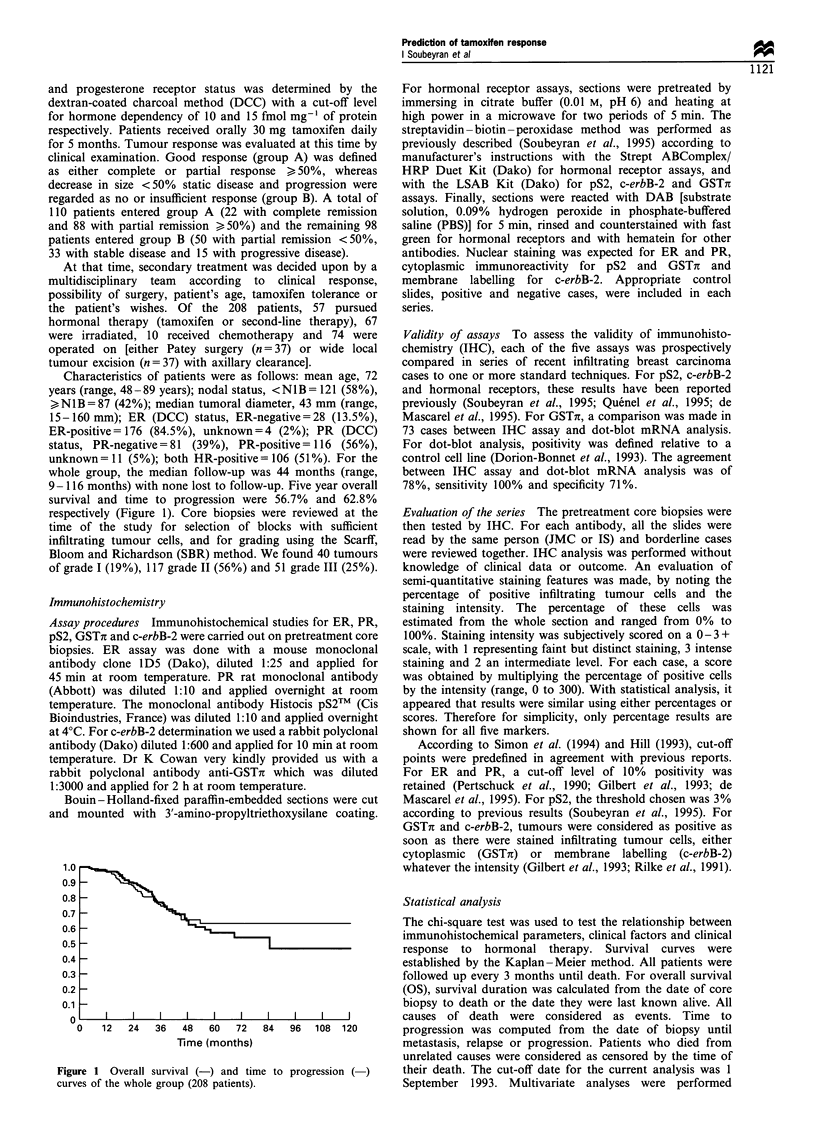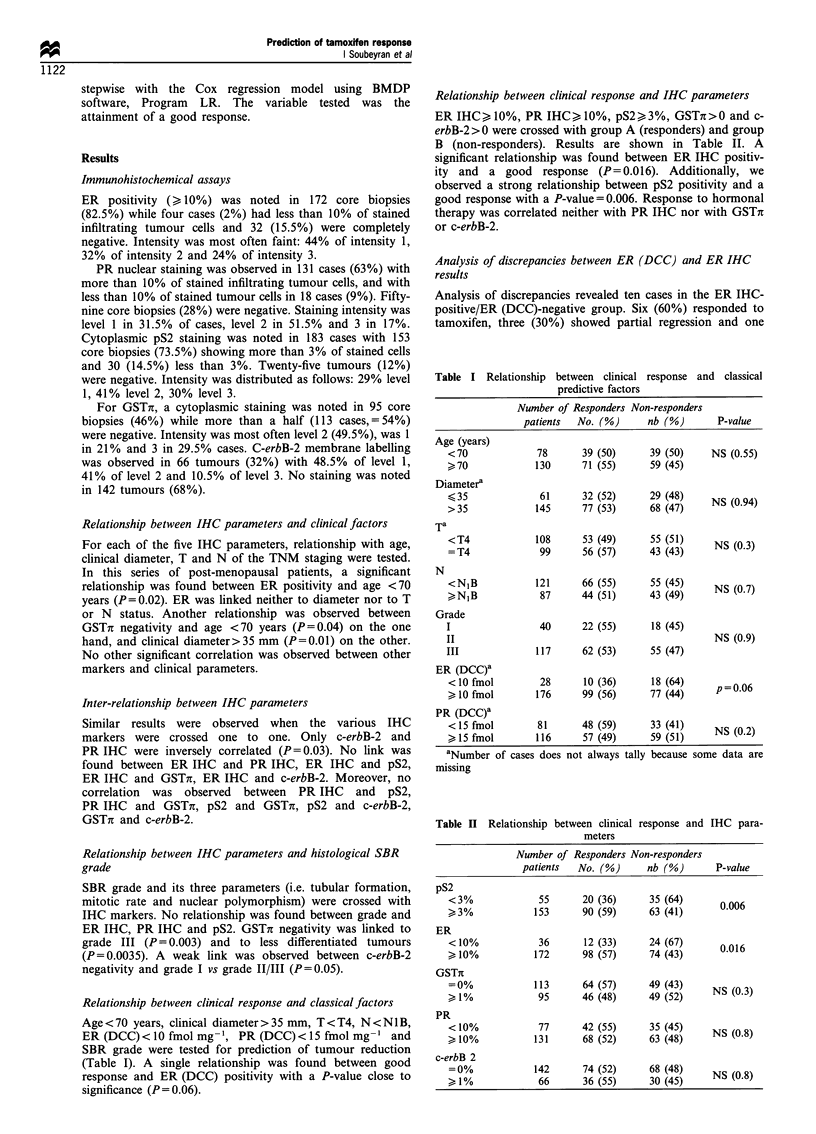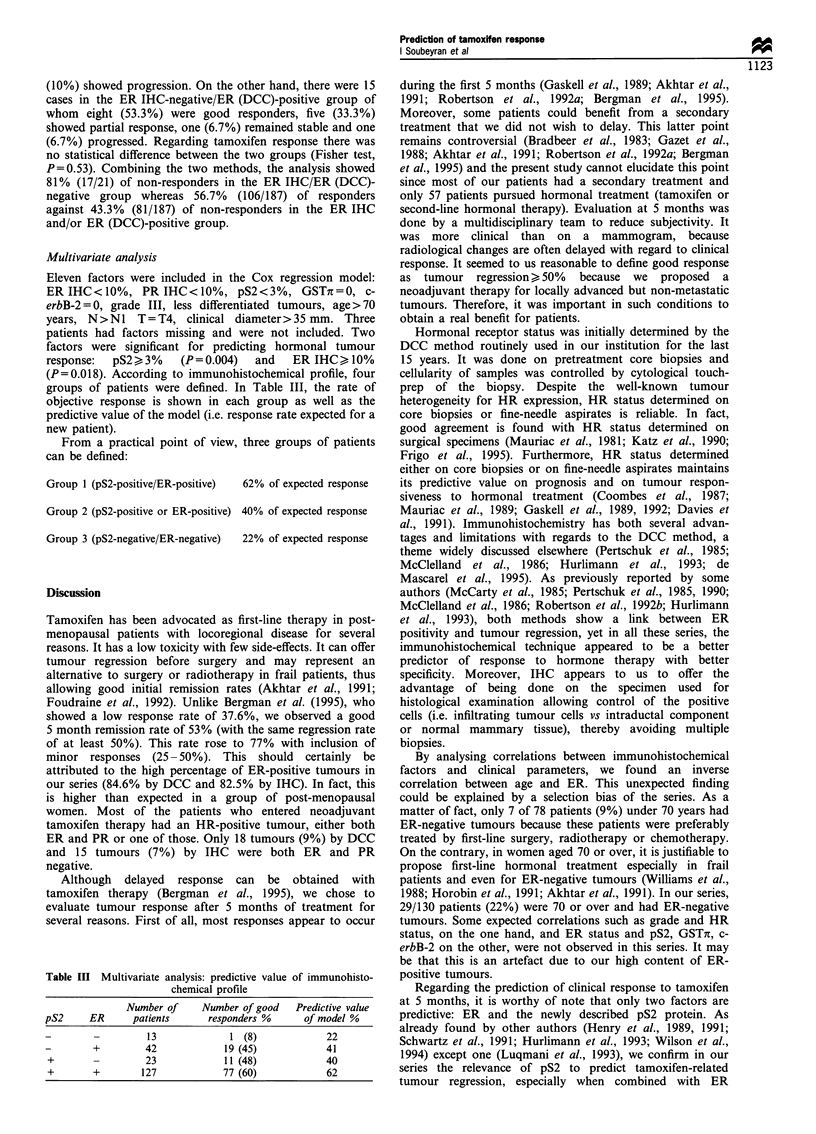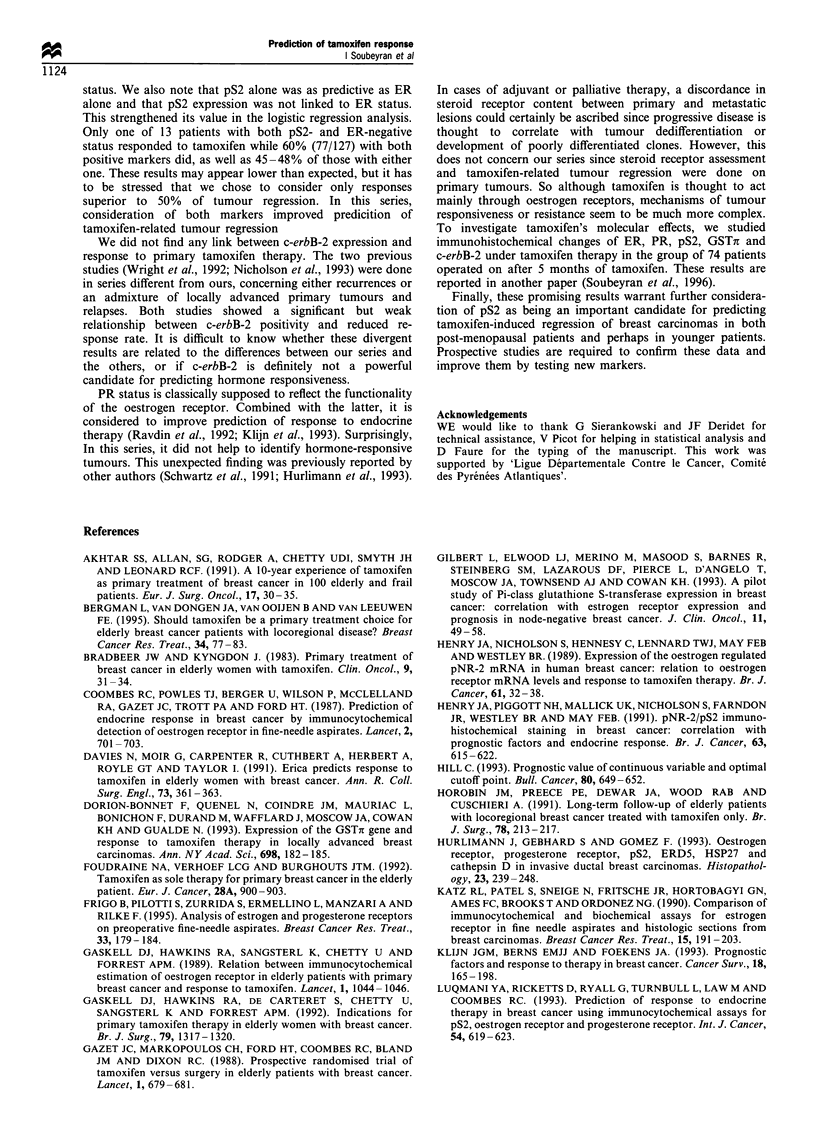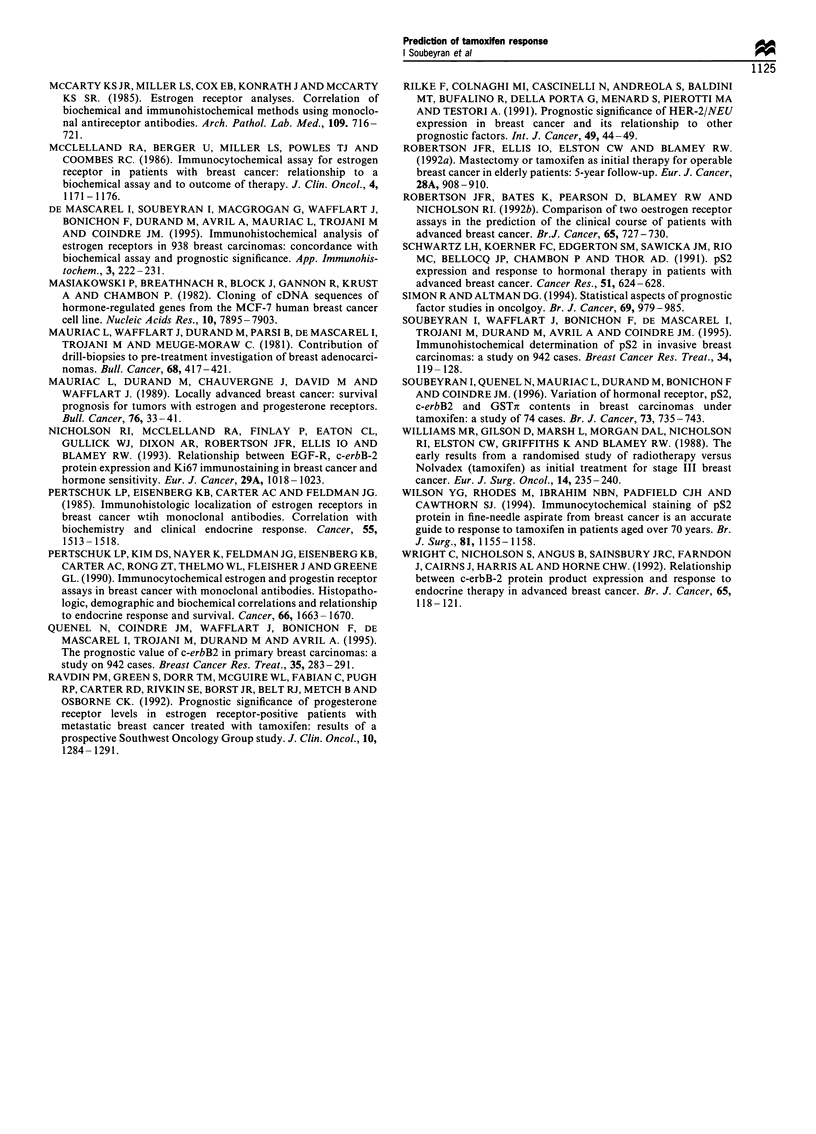# pS2 protein: a marker improving prediction of response to neoadjuvant tamoxifen in post-menopausal breast cancer patients.

**DOI:** 10.1038/bjc.1996.500

**Published:** 1996-10

**Authors:** I. Soubeyran, N. Quénel, J. M. Coindre, F. Bonichon, M. Durand, J. Wafflart, L. Mauriac

**Affiliations:** Institut Bergonié, Comprehensive Cancer Center, Bordeaux, France.

## Abstract

Tamoxifen as sole initial therapy is gaining importance in the management of post-menopausal breast cancer patients. Age oestrogen (ER) and progesterone (PR) receptor status are accurately considered to select patients for hormonal treatment. However, additional markers are needed. By immunohistochemistry (IHC), we studied tumour expression of ER, PR, pS2, c-erbB-2 and glutathione S-transferase pi (GST pi) on initial core biopsies of 208 post-menopausal patients with a non-metastatic invasive ductal carcinoma, treated by neoadjuvant tamoxifen therapy. A good response to tamoxifen was defined as tumoral regression > or = 50% (110 patients). Relationship between response and age, tumour size, T, N, histological grade, ER and PR contents evaluated by radioimmunoassay, ER, PR, pS2, c-erbB-2 and GST pi expression evaluated by IHC were studied. Univariate and multivariate analysis showed that tumoral regression was linked only to pS2 (P = 0.004) and ER (P = 0.018) IHC expression. According to the immunohistochemical profile, three groups could be defined: pS2- and ER-positive tumours, pS2- or ER-positive tumours and pS2- and ER-negative tumours with response rates of 60%, 45% and 8% respectively. Although prospective studies are needed to confirm these results, we conclude that pS2 and ER immunohistochemical status are useful tools for predicting tumour regression with neoadjuvant tamoxifen in post-menopausal breast carcinoma patients.


British Journal of Cancer (1996) 74, 1120-1125
© 1996 Stockton Press All rights reserved 0007-0920/96 $12.00

### pS2 protein: a marker improving prediction of response to neoadjuvant tamoxifen in post-menopausal breast cancer patients

I Soubeyran[1], N Quénel[1], J-M Coindre[1,2], F Bonichon[1], M Durand[1], J Wafflart[1] and L Mauriac[1]

[1]*Institut Bergonié, Comprehensive Cancer Center, 180 rue de Saint Genès, 33076 Bordeaux, France;* [2]*Université de Bordeaux II, Bordeaux, France.*

**Summary** Tamoxifen as sole initial therapy is gaining importance in the management of post-menopausal breast cancer patients. Age, oestrogen (ER) and progesterone (PR) receptor status are accurately considered to select patients for hormonal treatment. However, additional markers are needed. By immunohistochemistry (IHC), we studied tumour expression of ER, PR, pS2, c-*erb*B-2 and glutathione S-transferase $\pi$ (GST$\pi$) on initial core biopsies of 208 post-menopausal patients with a non-metastatic invasive ductal carcinoma, treated by neoadjuvant tamoxifen therapy. A good response to tamoxifen was defined as tumoral regression $\geqslant$50% (110 patients). Relationship between response and age, tumour size, T, N, histological grade, ER and PR contents evaluated by radioimmunoassay, ER, PR, pS2, c-*erb*B-2 and GST$\pi$ expression evaluated by IHC were studied. Univariate and multivariate analysis showed that tumoral regression was linked only to pS2 ($P$=0.004) and ER ($P$=0.018) IHC expression. According to the immunohistochemical profile, three groups could be defined: pS2- and ER-positive tumours, pS2- or ER-positive tumours and pS2- and ER-negative tumours with response rates of 60%, 45% and 8% respectively. Although prospective studies are needed to confirm these results, we conclude that pS2 and ER immunohistochemical status are useful tools for predicting tumour regression with neoadjuvant tamoxifen in post-menopausal breast carcinoma patients.

**Keywords:** breast neoplasm; tamoxifen; oestrogen receptor; progesterone receptor; pS2

The incidence of breast neoplasm increases with age. One-third of cases affect women older than 65 years. However, management of breast carcinomas in the elderly remains controversial. While some authors defend surgical treatment (Robertson *et al.*, 1992*a*), others have demonstrated the advantage of tamoxifen as sole initial therapy (Gazet *et al.*, 1988; Akhtar *et al.*, 1991; Horobin *et al.*, 1991; Foudraine *et al.*, 1992; Bergman *et al.*, 1995). In two randomised studies (Gazet *et al.*, 1988; Robertson *et al.*, 1992*a*), no difference in survival was found between the surgical and tamoxifen groups. Robertson *et al* observed only a better locoregional control in the mastectomy group, although not statistically significant. In fact, in elderly and frail patients, shorter life expectancy, concomitant diseases and increased surgery risk are prominent factors determining treatment choice. However, there is no precise way of knowing which patients can be treated with tamoxifen only as primary treatment. Until now, age and hormonal receptor status have been the main factors for identifying patients with hormone-sensitive tumours. Yet, in post-menopausal cohorts of breast cancer patients, in spite of a high proportion of ER-positive tumours, 30% to 60% of patients fail to respond to primary tamoxifen therapy (Gaskell *et al.*, 1989; Akhtar *et al.*, 1991; Horobin *et al.*, 1991; Robertson *et al.*, 1992*a*; Foudraine *et al.*, 1992; Bergman *et al.*, 1995). So it seems essential to look for additional markers of hormone responsiveness.

New insights into immunohistochemical techniques and new antibodies available make retrospective studies possible. In addition to ER and PR, new proteins more or less linked to hormonal receptors (HRs) have recently been isolated. Their role as prognostic factors and hormone-responsiveness markers has been suggested. Among them, we decided to examine pS2 protein, glutathione S-transferase $\pi$ (GST$\pi$) and c-*erb*B-2 oncogene. pS2 protein was first identified in the human breast cancer cell line MCF-7 in response to oestrogen stimulation (Masiakowski *et al.*, 1982). Recently, it has been proposed as a candidate for prediction of hormone-related tumour regression (Henry *et al.*, 1989; 1991; Schwartz *et al.*, 1991; Hurlimann *et al.*, 1993; Wilson *et al.*, 1994). Similarly, c-*erb*B-2 proto-oncogene, whose prognostic value in breast carcinomas is still controversial, has recently been implicated in predicting hormone-related tumour regression (Wright *et al.*, 1992; Nicholson *et al.*, 1993). GST$\pi$ enzyme is thought to play a role in the intracellular detoxification of a wide range of xenobiotics and anti-neoplastic drugs. In a preliminary study using dot-blot mRNA hybridisation, we found a link between response to tamoxifen therapy and low levels of GST$\pi$ mRNA (Dorion-Bonnet *et al.*, 1993).

For these reasons, we have studied immunohistochemical expression of ER, PR, pS2, GST$\pi$ and c-*erb*B-2 in a series of 208 non-metastatic post-menopausal patients with primary invasive breast carcinomas first treated in our centre by neoadjuvant tamoxifen therapy and evaluated for tumour regression.

### Materials and methods

### *Patients and tumours*

From 1984 to 1990, 2835 new patients with primary non-metastatic invasive ductal breast carcinomas were treated in our Comprehensive Cancer Center. Among them, 251 post-menopausal patients were first treated by neoadjuvant hormonal therapy (tamoxifen) for 5 months, for locally advanced HR-positive tumours. Of these 251 women, 43 were excluded: 26 because of insufficient histological material for IHC assays, two because of a bilateral tumour, one for concomitant chemotherapy and four owing to the patient's refusal to pursue hormonal therapy. Additionally, six patients were lost to follow-up and four died of intercurrent disease within the first 2 months. Patients who died from cancer or had a secondary treatment for progressive disease during this period were considered in progression and retained for the study.

All the 208 remaining patients underwent a core biopsy before treatment. At the time of diagnosis, patients were staged according to the UICC TNM classification. The two largest tumour diameters were clinically measured. Oestrogen

Correspondence: I Soubeyran, Institut Bergonié, Laboratoire d'Anatomie Pathologique, 180 rue de Saint Genès, 33076 Bordeaux cedex, France
Received 6 November 1995; revised 26 March 1996; accepted 29 April 1996

and progesterone receptor status was determined by the dextran-coated charcoal method (DCC) with a cut-off level for hormone dependency of 10 and 15 fmol $mg^{-1}$ of protein respectively. Patients received orally 30 mg tamoxifen daily for 5 months. Tumour response was evaluated at this time by clinical examination. Good response (group A) was defined as either complete or partial response $\geqslant 50\%$, whereas decrease in size $<50\%$ static disease and progression were regarded as no or insufficient response (group B). A total of 110 patients entered group A (22 with complete remission and 88 with partial remission $\geqslant 50\%$) and the remaining 98 patients entered group B (50 with partial remission $<50\%$, 33 with stable disease and 15 with progressive disease).

At that time, secondary treatment was decided upon by a multidisciplinary team according to clinical response, possibility of surgery, patient's age, tamoxifen tolerance or the patient's wishes. Of the 208 patients, 57 pursued hormonal therapy (tamoxifen or second-line therapy), 67 were irradiated, 10 received chemotherapy and 74 were operated on [either Patey surgery ($n=37$) or wide local tumour excision ($n=37$) with axillary clearance].

Characteristics of patients were as follows: mean age, 72 years (range, 48–89 years); nodal status, <N1B = 121 (58%), ≥N1B = 87 (42%); median tumoral diameter, 43 mm (range, 15–160 mm); ER (DCC) status, ER-negative = 28 (13.5%), ER-positive = 176 (84.5%), unknown = 4 (2%); PR (DCC) status, PR-negative = 81 (39%), PR-positive = 116 (56%), unknown = 11 (5%); both HR-positive = 106 (51%). For the whole group, the median follow-up was 44 months (range, 9–116 months) with none lost to follow-up. Five year overall survival and time to progression were 56.7% and 62.8% respectively (Figure 1). Core biopsies were reviewed at the time of the study for selection of blocks with sufficient infiltrating tumour cells, and for grading using the Scarff, Bloom and Richardson (SBR) method. We found 40 tumours of grade I (19%), 117 grade II (56%) and 51 grade III (25%).

### *Immunohistochemistry*

*Assay procedures* Immunohistochemical studies for ER, PR, pS2, GSTπ and c-*erb*B-2 were carried out on pretreatment core biopsies. ER assay was done with a mouse monoclonal antibody clone 1D5 (Dako), diluted 1:25 and applied for 45 min at room temperature. PR rat monoclonal antibody (Abbott) was diluted 1:10 and applied overnight at room temperature. The monoclonal antibody Histocis pS2™ (Cis Bioindustries, France) was diluted 1:10 and applied overnight at 4°C. For c-*erb*B-2 determination we used a rabbit polyclonal antibody (Dako) diluted 1:600 and applied for 10 min at room temperature. Dr K Cowan very kindly provided us with a rabbit polyclonal antibody anti-GSTπ which was diluted 1:3000 and applied for 2 h at room temperature.

Bouin–Holland-fixed paraffin-embedded sections were cut and mounted with 3′-amino-propyltriethoxysilane coating.

**Figure 1** Overall survival (—) and time to progression (—) curves of the whole group (208 patients).

For hormonal receptor assays, sections were pretreated by immersing in citrate buffer (0.01 M, pH 6) and heating at high power in a microwave for two periods of 5 min. The streptavidin–biotin–peroxidase method was performed as previously described (Soubeyran *et al.*, 1995) according to manufacturer's instructions with the Strept ABComplex/HRP Duet Kit (Dako) for hormonal receptor assays, and with the LSAB Kit (Dako) for pS2, c-*erb*B-2 and GSTπ assays. Finally, sections were reacted with DAB [substrate solution, 0.09% hydrogen peroxide in phosphate-buffered saline (PBS)] for 5 min, rinsed and counterstained with fast green for hormonal receptors and with hematein for other antibodies. Nuclear staining was expected for ER and PR, cytoplasmic immunoreactivity for pS2 and GSTπ and membrane labelling for c-*erb*B-2. Appropriate control slides, positive and negative cases, were included in each series.

*Validity of assays* To assess the validity of immunohistochemistry (IHC), each of the five assays was prospectively compared in series of recent infiltrating breast carcinoma cases to one or more standard techniques. For pS2, c-*erb*B-2 and hormonal receptors, these results have been reported previously (Soubeyran *et al.*, 1995; Quénel *et al.*, 1995; de Mascarel *et al.*, 1995). For GSTπ, a comparison was made in 73 cases between IHC assay and dot-blot mRNA analysis. For dot-blot analysis, positivity was defined relative to a control cell line (Dorion-Bonnet *et al.*, 1993). The agreement between IHC assay and dot-blot mRNA analysis was of 78%, sensitivity 100% and specificity 71%.

*Evaluation of the series* The pretreatment core biopsies were then tested by IHC. For each antibody, all the slides were read by the same person (JMC or IS) and borderline cases were reviewed together. IHC analysis was performed without knowledge of clinical data or outcome. An evaluation of semi-quantitative staining features was made, by noting the percentage of positive infiltrating tumour cells and the staining intensity. The percentage of these cells was estimated from the whole section and ranged from 0% to 100%. Staining intensity was subjectively scored on a 0–3+ scale, with 1 representing faint but distinct staining, 3 intense staining and 2 an intermediate level. For each case, a score was obtained by multiplying the percentage of positive cells by the intensity (range, 0 to 300). With statistical analysis, it appeared that results were similar using either percentages or scores. Therefore for simplicity, only percentage results are shown for all five markers.

According to Simon *et al.* (1994) and Hill (1993), cut-off points were predefined in agreement with previous reports. For ER and PR, a cut-off level of 10% positivity was retained (Pertschuck *et al.*, 1990; Gilbert *et al.*, 1993; de Mascarel *et al.*, 1995). For pS2, the threshold chosen was 3% according to previous results (Soubeyran *et al.*, 1995). For GSTπ and c-*erb*B-2, tumours were considered as positive as soon as there were stained infiltrating tumour cells, either cytoplasmic (GSTπ) or membrane labelling (c-*erb*B-2) whatever the intensity (Gilbert *et al.*, 1993; Rilke *et al.*, 1991).

### *Statistical analysis*

The chi-square test was used to test the relationship between immunohistochemical parameters, clinical factors and clinical response to hormonal therapy. Survival curves were established by the Kaplan–Meier method. All patients were followed up every 3 months until death. For overall survival (OS), survival duration was calculated from the date of core biopsy to death or the date they were last known alive. All causes of death were considered as events. Time to progression was computed from the date of biopsy until metastasis, relapse or progression. Patients who died from unrelated causes were considered as censored by the time of their death. The cut-off date for the current analysis was 1 September 1993. Multivariate analyses were performed stepwise with the Cox regression model using BMDP software, Program LR. The variable tested was the attainment of a good response.

### Results

*Immunohistochemical assays*

ER positivity (⩾10%) was noted in 172 core biopsies (82.5%) while four cases (2%) had less than 10% of stained infiltrating tumour cells and 32 (15.5%) were completely negative. Intensity was most often faint: 44% of intensity 1, 32% of intensity 2 and 24% of intensity 3.

PR nuclear staining was observed in 131 cases (63%) with more than 10% of stained infiltrating tumour cells, and with less than 10% of stained tumour cells in 18 cases (9%). Fifty-nine core biopsies (28%) were negative. Staining intensity was level 1 in 31.5% of cases, level 2 in 51.5% and 3 in 17%. Cytoplasmic pS2 staining was noted in 183 cases with 153 core biopsies (73.5%) showing more than 3% of stained cells and 30 (14.5%) less than 3%. Twenty-five tumours (12%) were negative. Intensity was distributed as follows: 29% level 1, 41% level 2, 30% level 3.

For GSTπ, a cytoplasmic staining was noted in 95 core biopsies (46%) while more than a half (113 cases, = 54%) were negative. Intensity was most often level 2 (49.5%), was 1 in 21% and 3 in 29.5% cases. C-*erb*B-2 membrane labelling was observed in 66 tumours (32%) with 48.5% of level 1, 41% of level 2 and 10.5% of level 3. No staining was noted in 142 tumours (68%).

*Relationship between IHC parameters and clinical factors*

For each of the five IHC parameters, relationship with age, clinical diameter, T and N of the TNM staging were tested. In this series of post-menopausal patients, a significant relationship was found between ER positivity and age <70 years ($P$=0.02). ER was linked neither to diameter nor to T or N status. Another relationship was observed between GSTπ negativity and age <70 years ($P$=0.04) on the one hand, and clinical diameter>35 mm ($P$=0.01) on the other. No other significant correlation was observed between other markers and clinical parameters.

*Inter-relationship between IHC parameters*

Similar results were observed when the various IHC markers were crossed one to one. Only c-*erb*B-2 and PR IHC were inversely correlated ($P$=0.03). No link was found between ER IHC and PR IHC, ER IHC and pS2, ER IHC and GSTπ, ER IHC and c-*erb*B-2. Moreover, no correlation was observed between PR IHC and pS2, PR IHC and GSTπ, pS2 and GSTπ, pS2 and c-*erb*B-2, GSTπ and c-*erb*B-2.

*Relationship between IHC parameters and histological SBR grade*

SBR grade and its three parameters (i.e. tubular formation, mitotic rate and nuclear polymorphism) were crossed with IHC markers. No relationship was found between grade and ER IHC, PR IHC and pS2. GSTπ negativity was linked to grade III ($P$=0.003) and to less differentiated tumours ($P$=0.0035). A weak link was observed between c-*erb*B-2 negativity and grade I *vs* grade II/III ($P$=0.05).

*Relationship between clinical response and classical factors*

Age<70 years, clinical diameter>35 mm, T<T4, N<N1B, ER (DCC)<10 fmol mg$^{-1}$, PR (DCC)<15 fmol mg$^{-1}$ and SBR grade were tested for prediction of tumour reduction (Table I). A single relationship was found between good response and ER (DCC) positivity with a $P$-value close to significance ($P$=0.06).

*Relationship between clinical response and IHC parameters*

ER IHC⩾10%, PR IHC⩾10%, pS2⩾3%, GSTπ>0 and c-*erb*B-2>0 were crossed with group A (responders) and group B (non-responders). Results are shown in Table II. A significant relationship was found between ER IHC positivity and a good response ($P$=0.016). Additionally, we observed a strong relationship between pS2 positivity and a good response with a $P$-value=0.006. Response to hormonal therapy was correlated neither with PR IHC nor with GSTπ or c-*erb*B-2.

*Analysis of discrepancies between ER (DCC) and ER IHC results*

Analysis of discrepancies revealed ten cases in the ER IHC-positive/ER (DCC)-negative group. Six (60%) responded to tamoxifen, three (30%) showed partial regression and one

**Table I** Relationship between clinical response and classical predictive factors

| | *Number of patients* | *Responders No. (%)* | *Non-responders nb (%)* | P-*value* |
|---|---|---|---|---|
| Age (years) | | | | |
| <70 | 78 | 39 (50) | 39 (50) | NS (0.55) |
| ⩾70 | 130 | 71 (55) | 59 (45) | |
| Diameter[a] | | | | |
| ⩽35 | 61 | 32 (52) | 29 (48) | NS (0.94) |
| >35 | 145 | 77 (53) | 68 (47) | |
| T[a] | | | | |
| <T4 | 108 | 53 (49) | 55 (51) | NS (0.3) |
| =T4 | 99 | 56 (57) | 43 (43) | |
| N | | | | |
| <$N_1$B | 121 | 66 (55) | 55 (45) | NS (0.7) |
| ⩾$N_1$B | 87 | 44 (51) | 43 (49) | |
| Grade | | | | |
| I | 40 | 22 (55) | 18 (45) | |
| II | | | | NS (0.9) |
| III | 117 | 62 (53) | 55 (47) | |
| ER (DCC)[a] | | | | |
| <10 fmol | 28 | 10 (36) | 18 (64) | $p$=0.06 |
| ⩾10 fmol | 176 | 99 (56) | 77 (44) | |
| PR (DCC)[a] | | | | |
| <15 fmol | 81 | 48 (59) | 33 (41) | NS (0.2) |
| ⩾15 fmol | 116 | 57 (49) | 59 (51) | |

[a]Number of cases does not always tally because some data are missing

**Table II** Relationship between clinical response and IHC parameters

| | *Number of patients* | *Responders No. (%)* | *Non-responders nb (%)* | P-*value* |
|---|---|---|---|---|
| pS2 | | | | |
| <3% | 55 | 20 (36) | 35 (64) | 0.006 |
| ⩾3% | 153 | 90 (59) | 63 (41) | |
| ER | | | | |
| <10% | 36 | 12 (33) | 24 (67) | 0.016 |
| ⩾10% | 172 | 98 (57) | 74 (43) | |
| GSTπ | | | | |
| =0% | 113 | 64 (57) | 49 (43) | NS (0.3) |
| ⩾1% | 95 | 46 (48) | 49 (52) | |
| PR | | | | |
| <10% | 77 | 42 (55) | 35 (45) | NS (0.8) |
| ⩾10% | 131 | 68 (52) | 63 (48) | |
| c-*erb*B 2 | | | | |
| =0% | 142 | 74 (52) | 68 (48) | NS (0.8) |
| ⩾1% | 66 | 36 (55) | 30 (45) | |

(10%) showed progression. On the other hand, there were 15 cases in the ER IHC-negative/ER (DCC)-positive group of whom eight (53.3%) were good responders, five (33.3%) showed partial response, one (6.7%) remained stable and one (6.7%) progressed. Regarding tamoxifen response there was no statistical difference between the two groups (Fisher test, $P=0.53$). Combining the two methods, the analysis showed 81% (17/21) of non-responders in the ER IHC/ER (DCC)-negative group whereas 56.7% (106/187) of responders against 43.3% (81/187) of non-responders in the ER IHC and/or ER (DCC)-positive group.

### *Multivariate analysis*

Eleven factors were included in the Cox regression model: ER IHC < 10%, PR IHC < 10%, pS2 < 3%, GST$\pi=0$, c-*erb*B-2 = 0, grade III, less differentiated tumours, age > 70 years, N > N1 T = T4, clinical diameter > 35 mm. Three patients had factors missing and were not included. Two factors were significant for predicting hormonal tumour response: pS2 $\geqslant$ 3% ($P=0.004$) and ER IHC $\geqslant$ 10% ($P=0.018$). According to immunohistochemical profile, four groups of patients were defined. In Table III, the rate of objective response is shown in each group as well as the predictive value of the model (i.e. response rate expected for a new patient).

From a practical point of view, three groups of patients can be defined:

Group 1 (pS2-positive/ER-positive) 62% of expected response

Group 2 (pS2-positive or ER-positive) 40% of expected response

Group 3 (pS2-negative/ER-negative) 22% of expected response

### Discussion

Tamoxifen has been advocated as first-line therapy in post-menopausal patients with locoregional disease for several reasons. It has a low toxicity with few side-effects. It can offer tumour regression before surgery and may represent an alternative to surgery or radiotherapy in frail patients, thus allowing good initial remission rates (Akhtar *et al.*, 1991; Foudraine *et al.*, 1992). Unlike Bergman *et al.* (1995), who showed a low response rate of 37.6%, we observed a good 5 month remission rate of 53% (with the same regression rate of at least 50%). This rate rose to 77% with inclusion of minor responses (25–50%). This should certainly be attributed to the high percentage of ER-positive tumours in our series (84.6% by DCC and 82.5% by IHC). In fact, this is higher than expected in a group of post-menopausal women. Most of the patients who entered neoadjuvant tamoxifen therapy had an HR-positive tumour, either both ER and PR or one of those. Only 18 tumours (9%) by DCC and 15 tumours (7%) by IHC were both ER and PR negative.

Although delayed response can be obtained with tamoxifen therapy (Bergman *et al.*, 1995), we chose to evaluate tumour response after 5 months of treatment for several reasons. First of all, most responses appear to occur during the first 5 months (Gaskell *et al.*, 1989; Akhtar *et al.*, 1991; Robertson *et al.*, 1992*a*; Bergman *et al.*, 1995). Moreover, some patients could benefit from a secondary treatment that we did not wish to delay. This latter point remains controversial (Bradbeer *et al.*, 1983; Gazet *et al.*, 1988; Akhtar *et al.*, 1991; Robertson *et al.*, 1992*a*; Bergman *et al.*, 1995) and the present study cannot elucidate this point since most of our patients had a secondary treatment and only 57 patients pursued hormonal treatment (tamoxifen or second-line hormonal therapy). Evaluation at 5 months was done by a multidisciplinary team to reduce subjectivity. It was more clinical than on a mammogram, because radiological changes are often delayed with regard to clinical response. It seemed to us reasonable to define good response as tumour regression $\geqslant$ 50% because we proposed a neoadjuvant therapy for locally advanced but non-metastatic tumours. Therefore, it was important in such conditions to obtain a real benefit for patients.

Hormonal receptor status was initially determined by the DCC method routinely used in our institution for the last 15 years. It was done on pretreatment core biopsies and cellularity of samples was controlled by cytological touch-prep of the biopsy. Despite the well-known tumour heterogeneity for HR expression, HR status determined on core biopsies or fine-needle aspirates is reliable. In fact, good agreement is found with HR status determined on surgical specimens (Mauriac *et al.*, 1981; Katz *et al.*, 1990; Frigo *et al.*, 1995). Furthermore, HR status determined either on core biopsies or on fine-needle aspirates maintains its predictive value on prognosis and on tumour responsiveness to hormonal treatment (Coombes *et al.*, 1987; Mauriac *et al.*, 1989; Gaskell *et al.*, 1989, 1992; Davies *et al.*, 1991). Immunohistochemistry has both several advantages and limitations with regards to the DCC method, a theme widely discussed elsewhere (Pertschuk *et al.*, 1985; McClelland *et al.*, 1986; Hurlimann *et al.*, 1993; de Mascarel *et al.*, 1995). As previously reported by some authors (McCarty *et al.*, 1985; Pertschuk *et al.*, 1985, 1990; McClelland *et al.*, 1986; Robertson *et al.*, 1992*b*; Hurlimann *et al.*, 1993), both methods show a link between ER positivity and tumour regression, yet in all these series, the immunohistochemical technique appeared to be a better predictor of response to hormone therapy with better specificity. Moreover, IHC appears to us to offer the advantage of being done on the specimen used for histological examination allowing control of the positive cells (i.e. infiltrating tumour cells *vs* intraductal component or normal mammary tissue), thereby avoiding multiple biopsies.

By analysing correlations between immunohistochemical factors and clinical parameters, we found an inverse correlation between age and ER. This unexpected finding could be explained by a selection bias of the series. As a matter of fact, only 7 of 78 patients (9%) under 70 years had ER-negative tumours because these patients were preferably treated by first-line surgery, radiotherapy or chemotherapy. On the contrary, in women aged 70 or over, it is justifiable to propose first-line hormonal treatment especially in frail patients and even for ER-negative tumours (Williams *et al.*, 1988; Horobin *et al.*, 1991; Akhtar *et al.*, 1991). In our series, 29/130 patients (22%) were 70 or over and had ER-negative tumours. Some expected correlations such as grade and HR status, on the one hand, and ER status and pS2, GST$\pi$, c-*erb*B-2 on the other, were not observed in this series. It may be that this is an artefact due to our high content of ER-positive tumours.

Regarding the prediction of clinical response to tamoxifen at 5 months, it is worthy of note that only two factors are predictive: ER and the newly described pS2 protein. As already found by other authors (Henry *et al.*, 1989, 1991; Schwartz *et al.*, 1991; Hurlimann *et al.*, 1993; Wilson *et al.*, 1994) except one (Luqmani *et al.*, 1993), we confirm in our series the relevance of pS2 to predict tamoxifen-related tumour regression, especially when combined with ER

**Table III** Multivariate analysis: predictive value of immunohistochemical profile

| *pS2* | *ER* | *Number of patients* | *Number of good responders %* | *Predictive value of model %* |
|---|---|---|---|---|
| – | – | 13 | 1 (8) | 22 |
| – | + | 42 | 19 (45) | 41 |
| + | – | 23 | 11 (48) | 40 |
| + | + | 127 | 77 (60) | 62 |

status. We also note that pS2 alone was as predictive as ER alone and that pS2 expression was not linked to ER status. This strengthened its value in the logistic regression analysis. Only one of 13 patients with both pS2- and ER-negative status responded to tamoxifen while 60% (77/127) with both positive markers did, as well as 45–48% of those with either one. These results may appear lower than expected, but it has to be stressed that we chose to consider only responses superior to 50% of tumour regression. In this series, consideration of both markers improved predicition of tamoxifen-related tumour regression

We did not find any link between c-*erb*B-2 expression and response to primary tamoxifen therapy. The two previous studies (Wright *et al.*, 1992; Nicholson *et al.*, 1993) were done in series different from ours, concerning either recurrences or an admixture of locally advanced primary tumours and relapses. Both studies showed a significant but weak relationship between c-*erb*B-2 positivity and reduced response rate. It is difficult to know whether these divergent results are related to the differences between our series and the others, or if c-*erb*B-2 is definitely not a powerful candidate for predicting hormone responsiveness.

PR status is classically supposed to reflect the functionality of the oestrogen receptor. Combined with the latter, it is considered to improve prediction of response to endocrine therapy (Ravdin *et al.*, 1992; Klijn *et al.*, 1993). Surprisingly, In this series, it did not help to identify hormone-responsive tumours. This unexpected finding was previously reported by other authors (Schwartz *et al.*, 1991; Hurlimann *et al.*, 1993).

In cases of adjuvant or palliative therapy, a discordance in steroid receptor content between primary and metastatic lesions could certainly be ascribed since progressive disease is thought to correlate with tumour dedifferentiation or development of poorly differentiated clones. However, this does not concern our series since steroid receptor assessment and tamoxifen-related tumour regression were done on primary tumours. So although tamoxifen is thought to act mainly through oestrogen receptors, mechanisms of tumour responsiveness or resistance seem to be much more complex. To investigate tamoxifen's molecular effects, we studied immunohistochemical changes of ER, PR, pS2, GST$\pi$ and c-*erb*B-2 under tamoxifen therapy in the group of 74 patients operated on after 5 months of tamoxifen. These results are reported in another paper (Soubeyran *et al.*, 1996).

Finally, these promising results warrant further consideration of pS2 as being an important candidate for predicting tamoxifen-induced regression of breast carcinomas in both post-menopausal patients and perhaps in younger patients. Prospective studies are required to confirm these data and improve them by testing new markers.

**Acknowledgements**

WE would like to thank G Sierankowski and JF Deridet for technical assistance, V Picot for helping in statistical analysis and D Faure for the typing of the manuscript. This work was supported by 'Ligue Départementale Contre le Cancer, Comité des Pyrénées Atlantiques'.

### References

AKHTAR SS, ALLAN, SG, RODGER A, CHETTY UDI, SMYTH JH AND LEONARD RCF. (1991). A 10-year experience of tamoxifen as primary treatment of breast cancer in 100 elderly and frail patients. *Eur. J. Surg. Oncol.*, **17**, 30–35.

BERGMAN L, VAN DONGEN JA, VAN OOIJEN B AND VAN LEEUWEN FE. (1995). Should tamoxifen be a primary treatment choice for elderly breast cancer patients with locoregional disease? *Breast Cancer Res. Treat.*, **34**, 77–83.

BRADBEER JW AND KYNGDON J. (1983). Primary treatment of breast cancer in elderly women with tamoxifen. *Clin. Oncol.*, **9**, 31–34.

COOMBES RC, POWLES TJ, BERGER U, WILSON P, MCCLELLAND RA, GAZET JC, TROTT PA AND FORD HT. (1987). Prediction of endocrine response in breast cancer by immunocytochemical detection of oestrogen receptor in fine-needle aspirates. *Lancet*, **2**, 701–703.

DAVIES N, MOIR G, CARPENTER R, CUTHBERT A, HERBERT A, ROYLE GT AND TAYLOR I. (1991). Erica predicts response to tamoxifen in elderly women with breast cancer. *Ann. R. Coll. Surg. Engl.*, **73**, 361–363.

DORION-BONNET F, QUENEL N, COINDRE JM, MAURIAC L, BONICHON F, DURAND M, WAFFLARD J, MOSCOW JA, COWAN KH AND GUALDE N. (1993). Expression of the GST$\pi$ gene and response to tamoxifen therapy in locally advanced breast carcinomas. *Ann. NY Acad. Sci.*, **698**, 182–185.

FOUDRAINE NA, VERHOEF LCG AND BURGHOUTS JTM. (1992). Tamoxifen as sole therapy for primary breast cancer in the elderly patient. *Eur. J. Cancer*, **28A**, 900–903.

FRIGO B, PILOTTI S, ZURRIDA S, ERMELLINO L, MANZARI A AND RILKE F. (1995). Analysis of estrogen and progesterone receptors on preoperative fine-needle aspirates. *Breast Cancer Res. Treat.*, **33**, 179–184.

GASKELL DJ, HAWKINS RA, SANGSTERL K, CHETTY U AND FORREST APM. (1989). Relation between immunocytochemical estimation of oestrogen receptor in elderly patients with primary breast cancer and response to tamoxifen. *Lancet*, **1**, 1044–1046.

GASKELL DJ, HAWKINS RA, DE CARTERET S, CHETTY U, SANGSTERL K AND FORREST APM. (1992). Indications for primary tamoxifen therapy in elderly women with breast cancer. *Br. J. Surg.*, **79**, 1317–1320.

GAZET JC, MARKOPOULOS CH, FORD HT, COOMBES RC, BLAND JM AND DIXON RC. (1988). Prospective randomised trial of tamoxifen versus surgery in elderly patients with breast cancer. *Lancet*, **1**, 679–681.

GILBERT L, ELWOOD LJ, MERINO M, MASOOD S, BARNES R, STEINBERG SM, LAZAROUS DF, PIERCE L, D'ANGELO T, MOSCOW JA, TOWNSEND AJ AND COWAN KH. (1993). A pilot study of Pi-class glutathione S-transferase expression in breast cancer: correlation with estrogen receptor expression and prognosis in node-negative breast cancer. *J. Clin. Oncol.*, **11**, 49–58.

HENRY JA, NICHOLSON S, HENNESY C, LENNARD TWJ, MAY FEB AND WESTLEY BR. (1989). Expression of the oestrogen regulated pNR-2 mRNA in human breast cancer: relation to oestrogen receptor mRNA levels and response to tamoxifen therapy. *Br. J. Cancer*, **61**, 32–38.

HENRY JA, PIGGOTT NH, MALLICK UK, NICHOLSON S, FARNDON JR, WESTLEY BR AND MAY FEB. (1991). pNR-2/pS2 immunohistochemical staining in breast cancer: correlation with prognostic factors and endocrine response. *Br. J. Cancer*, **63**, 615–622.

HILL C. (1993). Prognostic value of continuous variable and optimal cutoff point. *Bull. Cancer*, **80**, 649–652.

HOROBIN JM, PREECE PE, DEWAR JA, WOOD RAB AND CUSCHIERI A. (1991). Long-term follow-up of elderly patients with locoregional breast cancer treated with tamoxifen only. *Br. J. Surg.*, **78**, 213–217.

HURLIMANN J, GEBHARD S AND GOMEZ F. (1993). Oestrogen receptor, progesterone receptor, pS2, ERD5, HSP27 and cathepsin D in invasive ductal breast carcinomas. *Histopathology*, **23**, 239–248.

KATZ RL, PATEL S, SNEIGE N, FRITSCHE JR, HORTOBAGYI GN, AMES FC, BROOKS T AND ORDONEZ NG. (1990). Comparison of immunocytochemical and biochemical assays for estrogen receptor in fine needle aspirates and histologic sections from breast carcinomas. *Breast Cancer Res. Treat.*, **15**, 191–203.

KLIJN JGM, BERNS EMJJ AND FOEKENS JA. (1993). Prognostic factors and response to therapy in breast cancer. *Cancer Surv.*, **18**, 165–198.

LUQMANI YA, RICKETTS D, RYALL G, TURNBULL L, LAW M AND COOMBES RC. (1993). Prediction of response to endocrine therapy in breast cancer using immunocytochemical assays for pS2, oestrogen receptor and progesterone receptor. *Int. J. Cancer*, **54**, 619–623.

McCarty KS Jr, Miller LS, Cox EB, Konrath J and McCarty KS Sr. (1985). Estrogen receptor analyses. Correlation of biochemical and immunohistochemical methods using monoclonal antireceptor antibodies. *Arch. Pathol. Lab. Med.*, **109**. 716–721.

McClelland RA, Berger U, Miller LS, Powles TJ and Coombes RC. (1986). Immunocytochemical assay for estrogen receptor in patients with breast cancer: relationship to a biochemical assay and to outcome of therapy. *J. Clin. Oncol.*, **4**, 1171–1176.

de Mascarel I, Soubeyran I, MacGrogan G, Wafflart J, Bonichon F, Durand M, Avril A, Mauriac L, Trojani M and Coindre JM. (1995). Immunohistochemical analysis of estrogen receptors in 938 breast carcinomas: concordance with biochemical assay and prognostic significance. *App. Immunohistochem.*, **3**, 222–231.

Masiakowski P, Breathnach R, Block J, Gannon R, Krust A and Chambon P. (1982). Cloning of cDNA sequences of hormone-regulated genes from the MCF-7 human breast cancer cell line. *Nucleic Acids Res.*, **10**, 7895–7903.

Mauriac L, Wafflart J, Durand M, Parsi B, de Mascarel I, Trojani M and Meuge-Moraw C. (1981). Contribution of drill-biopsies to pre-treatment investigation of breast adenocarcinomas. *Bull. Cancer*, **68**, 417–421.

Mauriac L, Durand M, Chauvergne J, David M and Wafflart J. (1989). Locally advanced breast cancer: survival prognosis for tumors with estrogen and progesterone receptors. *Bull. Cancer*, **76**, 33–41.

Nicholson RI, McClelland RA, Finlay P, Eaton CL, Gullick WJ, Dixon AR, Robertson JFR, Ellis IO and Blamey RW. (1993). Relationship between EGF-R, c-*erb*B-2 protein expression and Ki67 immunostaining in breast cancer and hormone sensitivity. *Eur. J. Cancer*, **29A**, 1018–1023.

Pertschuk LP, Eisenberg KB, Carter AC and Feldman JG. (1985). Immunohistologic localization of estrogen receptors in breast cancer wtih monoclonal antibodies. Correlation with biochemistry and clinical endocrine response. *Cancer*, **55**, 1513–1518.

Pertschuk LP, Kim DS, Nayer K, Feldman JG, Eisenberg KB, Carter AC, Rong ZT, Thelmo WL, Fleisher J and Greene GL. (1990). Immunocytochemical estrogen and progestin receptor assays in breast cancer with monoclonal antibodies. Histopathologic, demographic and biochemical correlations and relationship to endocrine response and survival. *Cancer*, **66**, 1663–1670.

Quenel N, Coindre JM, Wafflart J, Bonichon F, de Mascarel I, Trojani M, Durand M and Avril A. (1995). The prognostic value of c-*erb*B2 in primary breast carcinomas: a study on 942 cases. *Breast Cancer Res. Treat.*, **35**, 283–291.

Ravdin PM, Green S, Dorr TM, McGuire WL, Fabian C, Pugh RP, Carter RD, Rivkin SE, Borst JR, Belt RJ, Metch B and Osborne CK. (1992). Prognostic significance of progesterone receptor levels in estrogen receptor-positive patients with metastatic breast cancer treated with tamoxifen: results of a prospective Southwest Oncology Group study. *J. Clin. Oncol.*, **10**, 1284–1291.

Rilke F, Colnaghi MI, Cascinelli N, Andreola S, Baldini MT, Bufalino R, Della Porta G, Menard S, Pierotti MA and Testori A. (1991). Prognostic significance of HER-2/*NEU* expression in breast cancer and its relationship to other prognostic factors. *Int. J. Cancer*, **49**, 44–49.

Robertson JFR, Ellis IO, Elston CW and Blamey RW. (1992*a*). Mastectomy or tamoxifen as initial therapy for operable breast cancer in elderly patients: 5-year follow-up. *Eur. J. Cancer*, **28A**, 908–910.

Robertson JFR, Bates K, Pearson D, Blamey RW and Nicholson RI. (1992*b*). Comparison of two oestrogen receptor assays in the prediction of the clinical course of patients with advanced breast cancer. *Br.J. Cancer*, **65**, 727–730.

Schwartz LH, Koerner FC, Edgerton SM, Sawicka JM, Rio MC, Bellocq JP, Chambon P and Thor AD. (1991). pS2 expression and response to hormonal therapy in patients with advanced breast cancer. *Cancer Res.*, **51**, 624–628.

Simon R and Altman DG. (1994). Statistical aspects of prognostic factor studies in oncolgoy. *Br. J. Cancer*, **69**, 979–985.

Soubeyran I, Wafflart J, Bonichon F, de Mascarel I, Trojani M, Durand M, Avril A and Coindre JM. (1995). Immunohistochemical determination of pS2 in invasive breast carcinomas: a study on 942 cases. *Breast Cancer Res. Treat.*, **34**, 119–128.

Soubeyran I, Quenel N, Mauriac L, Durand M, Bonichon F and Coindre JM. (1996). Variation of hormonal receptor, pS2, c-*erb*B2 and GST$\pi$ contents in breast carcinomas under tamoxifen: a study of 74 cases. *Br. J. Cancer*, **73**, 735–743.

Williams MR, Gilson D, Marsh L, Morgan DAL, Nicholson RI, Elston CW, Griffiths K and Blamey RW. (1988). The early results from a randomised study of radiotherapy versus Nolvadex (tamoxifen) as initial treatment for stage III breast cancer. *Eur. J. Surg. Oncol.*, **14**, 235–240.

Wilson YG, Rhodes M, Ibrahim NBN, Padfield CJH and Cawthorn SJ. (1994). Immunocytochemical staining of pS2 protein in fine-needle aspirate from breast cancer is an accurate guide to response to tamoxifen in patients aged over 70 years. *Br. J. Surg.*, **81**, 1155–1158.

Wright C, Nicholson S, Angus B, Sainsbury JRC, Farndon J, Cairns J, Harris AL and Horne CHW. (1992). Relationship between c-erbB-2 protein product expression and response to endocrine therapy in advanced breast cancer. *Br. J. Cancer*, **65**, 118–121.